# Supplementary material for: Endarachne binghamiae Ameliorates Hepatic Steatosis, Obesity, and Blood Glucose via Modulation of Metabolic Pathways and Oxidative Stress
Source: Int J Mol Sci. 2025 May 26;26(11):5103. doi: 10.3390/ijms26115103 (PMC12154224; doi:10.3390/ijms26115103)
Supplement: Supplementary file 1 [file ijms-26-05103-s001.zip › Suplemental data-revised.pptx]

## Slide 1
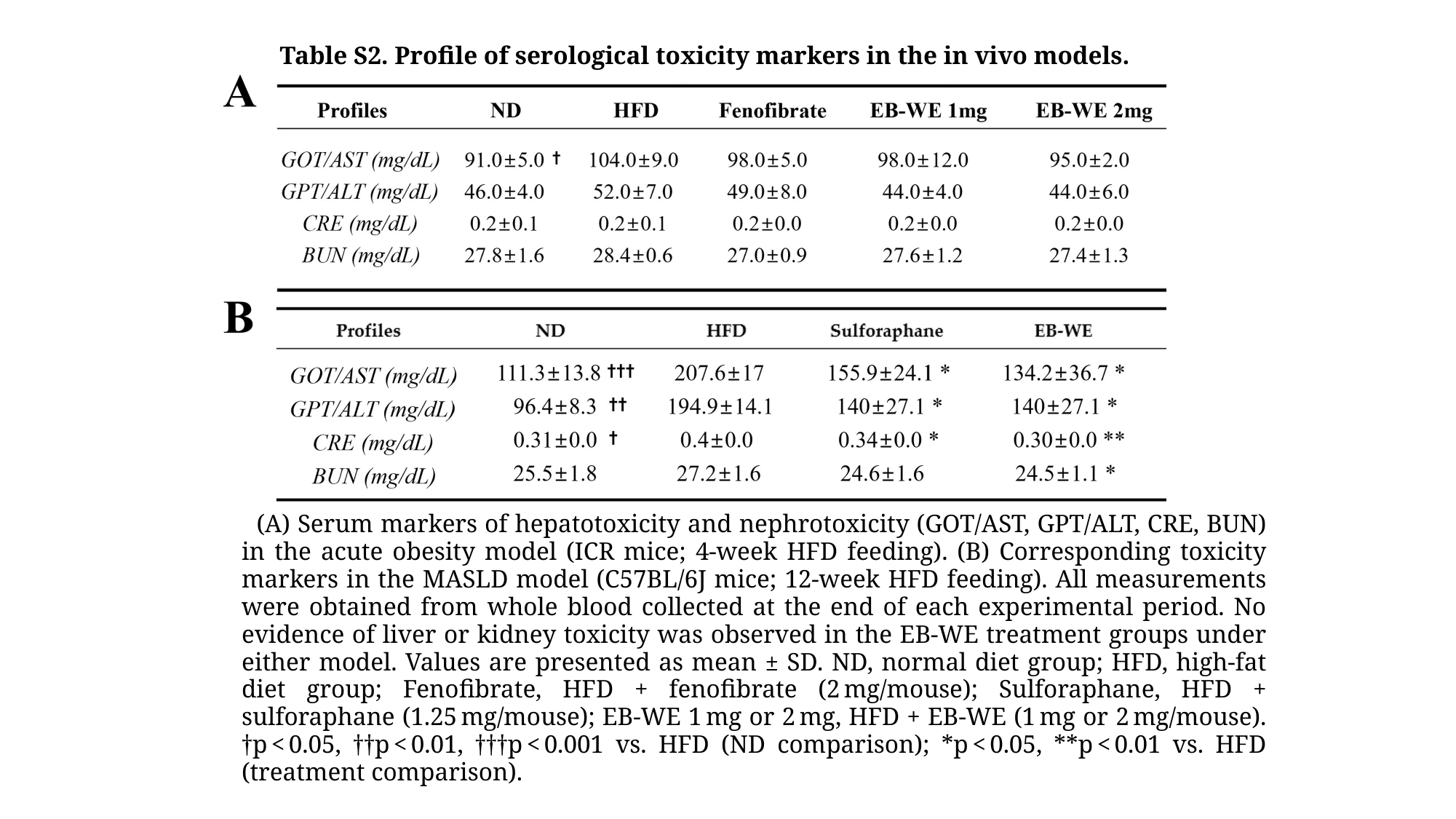

Table S2. Profile of serological toxicity markers in the in vivo models.
 (A) Serum markers of hepatotoxicity and nephrotoxicity (GOT/AST, GPT/ALT, CRE, BUN) in the acute obesity model (ICR mice; 4-week HFD feeding). (B) Corresponding toxicity markers in the MASLD model (C57BL/6J mice; 12-week HFD feeding). All measurements were obtained from whole blood collected at the end of each experimental period. No evidence of liver or kidney toxicity was observed in the EB-WE treatment groups under either model. Values are presented as mean ± SD. ND, normal diet group; HFD, high-fat diet group; Fenofibrate, HFD + fenofibrate (2 mg/mouse); Sulforaphane, HFD + sulforaphane (1.25 mg/mouse); EB-WE 1 mg or 2 mg, HFD + EB-WE (1 mg or 2 mg/mouse). †p < 0.05, ††p < 0.01, †††p < 0.001 vs. HFD (ND comparison); *p < 0.05, **p < 0.01 vs. HFD (treatment comparison).

## Slide 2
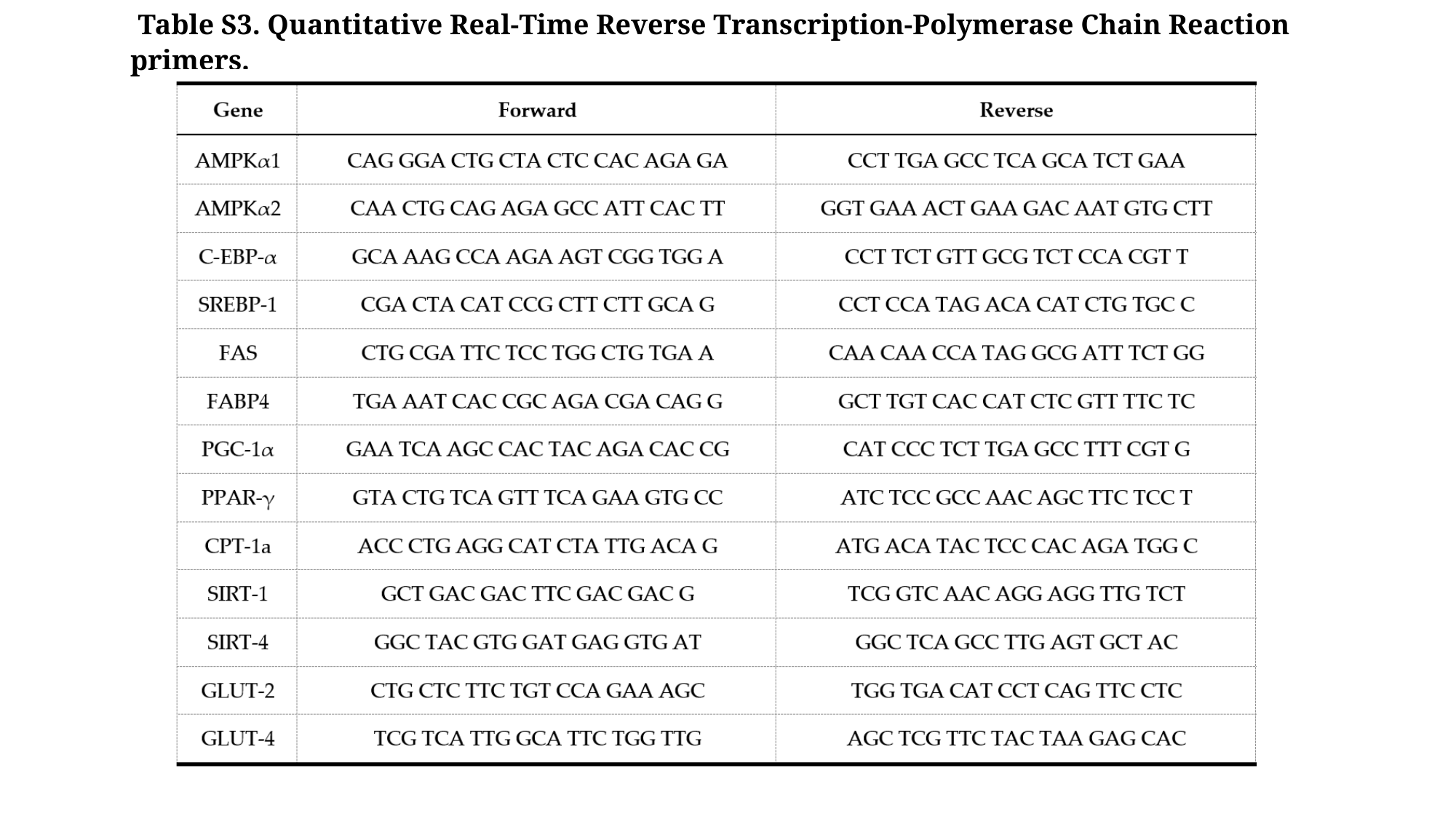

Table S3. Quantitative Real-Time Reverse Transcription-Polymerase Chain Reaction primers.

## Slide 3
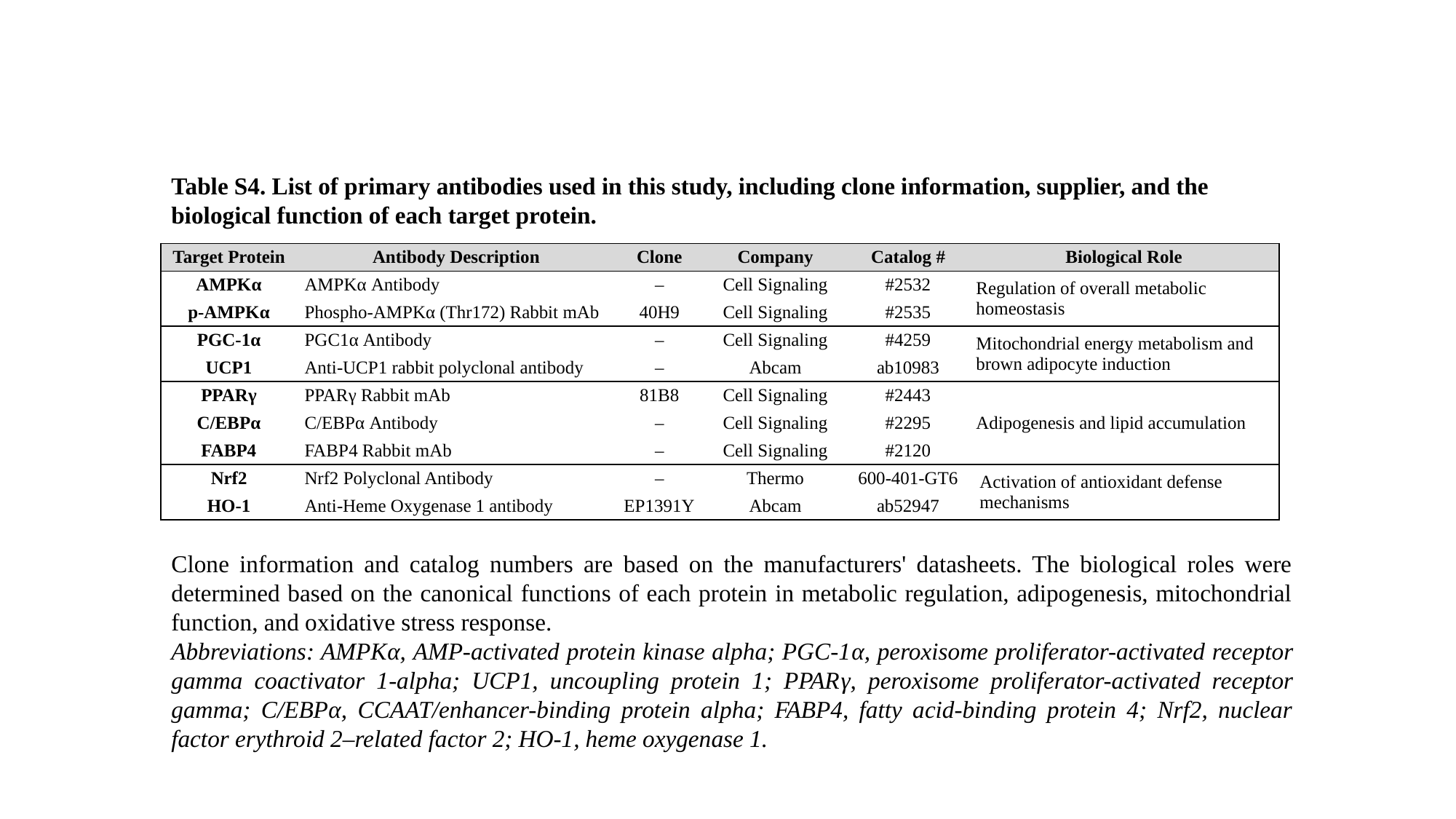

Table S4. List of primary antibodies used in this study, including clone information, supplier, and the biological function of each target protein.
Clone information and catalog numbers are based on the manufacturers' datasheets. The biological roles were determined based on the canonical functions of each protein in metabolic regulation, adipogenesis, mitochondrial function, and oxidative stress response.
Abbreviations: AMPKα, AMP-activated protein kinase alpha; PGC-1α, peroxisome proliferator-activated receptor gamma coactivator 1-alpha; UCP1, uncoupling protein 1; PPARγ, peroxisome proliferator-activated receptor gamma; C/EBPα, CCAAT/enhancer-binding protein alpha; FABP4, fatty acid-binding protein 4; Nrf2, nuclear factor erythroid 2–related factor 2; HO-1, heme oxygenase 1.
| Target Protein | Antibody Description | Clone | Company | Catalog # | Biological Role |
| --- | --- | --- | --- | --- | --- |
| AMPKα | AMPKα Antibody | – | Cell Signaling | #2532 | Regulation of overall metabolic homeostasis |
| p-AMPKα | Phospho-AMPKα (Thr172) Rabbit mAb | 40H9 | Cell Signaling | #2535 | Regulation of overall metabolic homeostasis |
| PGC-1α | PGC1α Antibody | – | Cell Signaling | #4259 | Mitochondrial energy metabolism and brown adipocyte induction |
| UCP1 | Anti-UCP1 rabbit polyclonal antibody | – | Abcam | ab10983 | |
| PPARγ | PPARγ Rabbit mAb | 81B8 | Cell Signaling | #2443 | Adipogenesis and lipid accumulation |
| C/EBPα | C/EBPα Antibody | – | Cell Signaling | #2295 | |
| FABP4 | FABP4 Rabbit mAb | – | Cell Signaling | #2120 | |
| Nrf2 | Nrf2 Polyclonal Antibody | – | Thermo | 600-401-GT6 | Activation of antioxidant defense mechanisms |
| HO-1 | Anti-Heme Oxygenase 1 antibody | EP1391Y | Abcam | ab52947 | |

## Slide 4
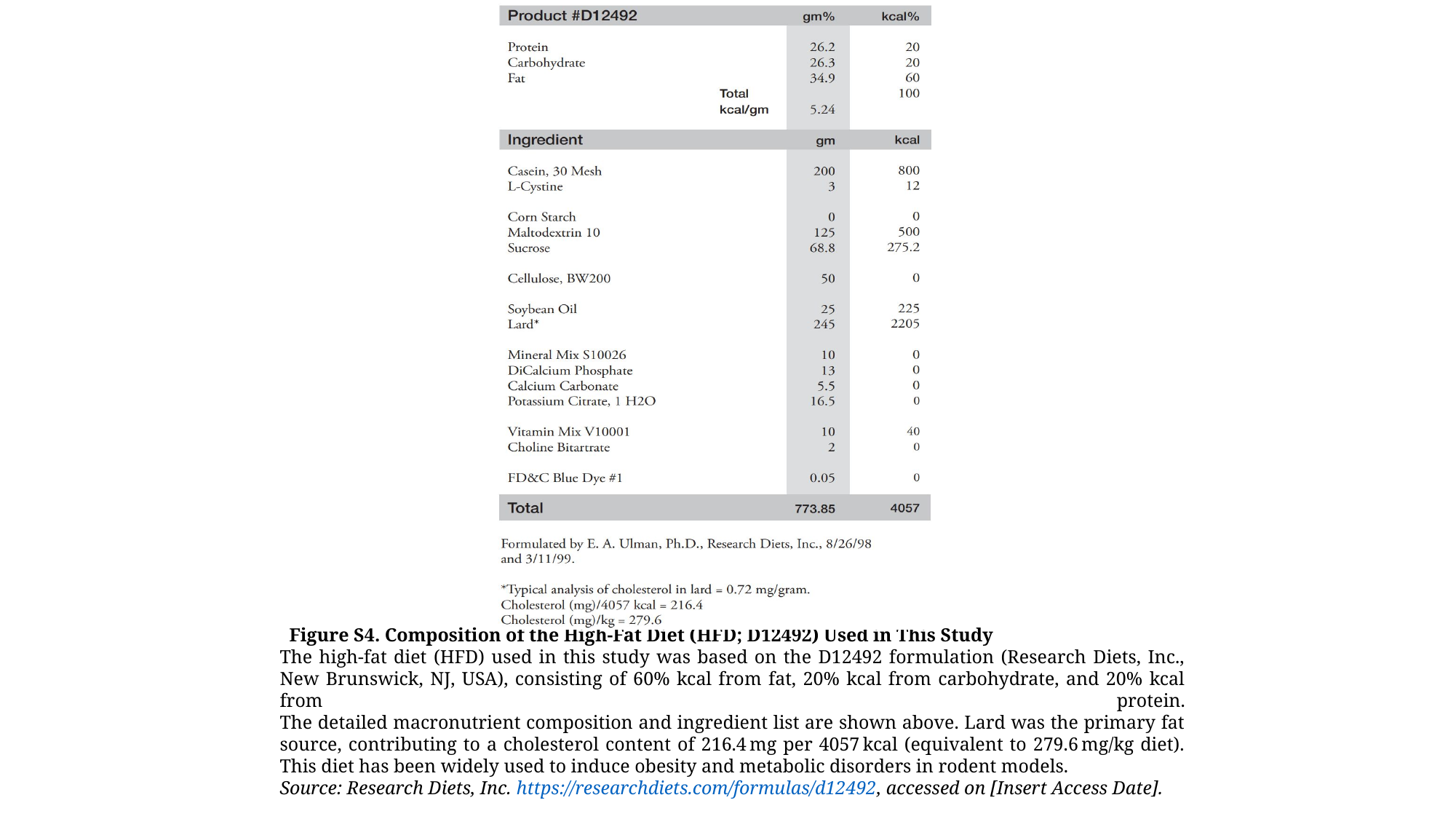

Figure S4. Composition of the High-Fat Diet (HFD; D12492) Used in This Study
The high-fat diet (HFD) used in this study was based on the D12492 formulation (Research Diets, Inc., New Brunswick, NJ, USA), consisting of 60% kcal from fat, 20% kcal from carbohydrate, and 20% kcal from protein.
The detailed macronutrient composition and ingredient list are shown above. Lard was the primary fat source, contributing to a cholesterol content of 216.4 mg per 4057 kcal (equivalent to 279.6 mg/kg diet).
This diet has been widely used to induce obesity and metabolic disorders in rodent models.
Source: Research Diets, Inc. https://researchdiets.com/formulas/d12492, accessed on [Insert Access Date].
